# Supplementary material for: Introducing synthetic thermostable RNase inhibitors to single-cell RNA-seq
Source: Nat Commun. 2024 Sep 27;15:8373. doi: 10.1038/s41467-024-52717-4 (PMC11437267; doi:10.1038/s41467-024-52717-4)
Supplement: Supplementary file 1 — Supplementary Information [file 41467_2024_52717_MOESM1_ESM.pdf]

nature communications

Supplementary Information for:
Introducing synthetic thermostable RNase inhibitors to single-cell RNA-seq

Joyce Carol Noble[1], Antonio Lentini[1], Michael Hagemann-Jensen[2], Rickard Sandberg[2], and Björn Reinius[1, #]

[1] Department of Medical Biochemistry and Biophysics, Karolinska Institutet, Stockholm, Sweden.

[2] Department of Cell and Molecular Biology, Karolinska Institutet, Stockholm, Sweden.

# Correspondence: bjorn.reinius@ki.se

The following information is included in this file:

Supplementary Figures 1–9

Supplementary Note 1: Smart-seq2 with SEQURNA thermostable RNase inhibitor

Supplementary Note 2: Smart-seq3 with SEQURNA thermostable RNase inhibitor

Supplementary Note 3: Smart-seq3xpress with SEQURNA thermostable RNase inhibitor

Supplementary Figure 1

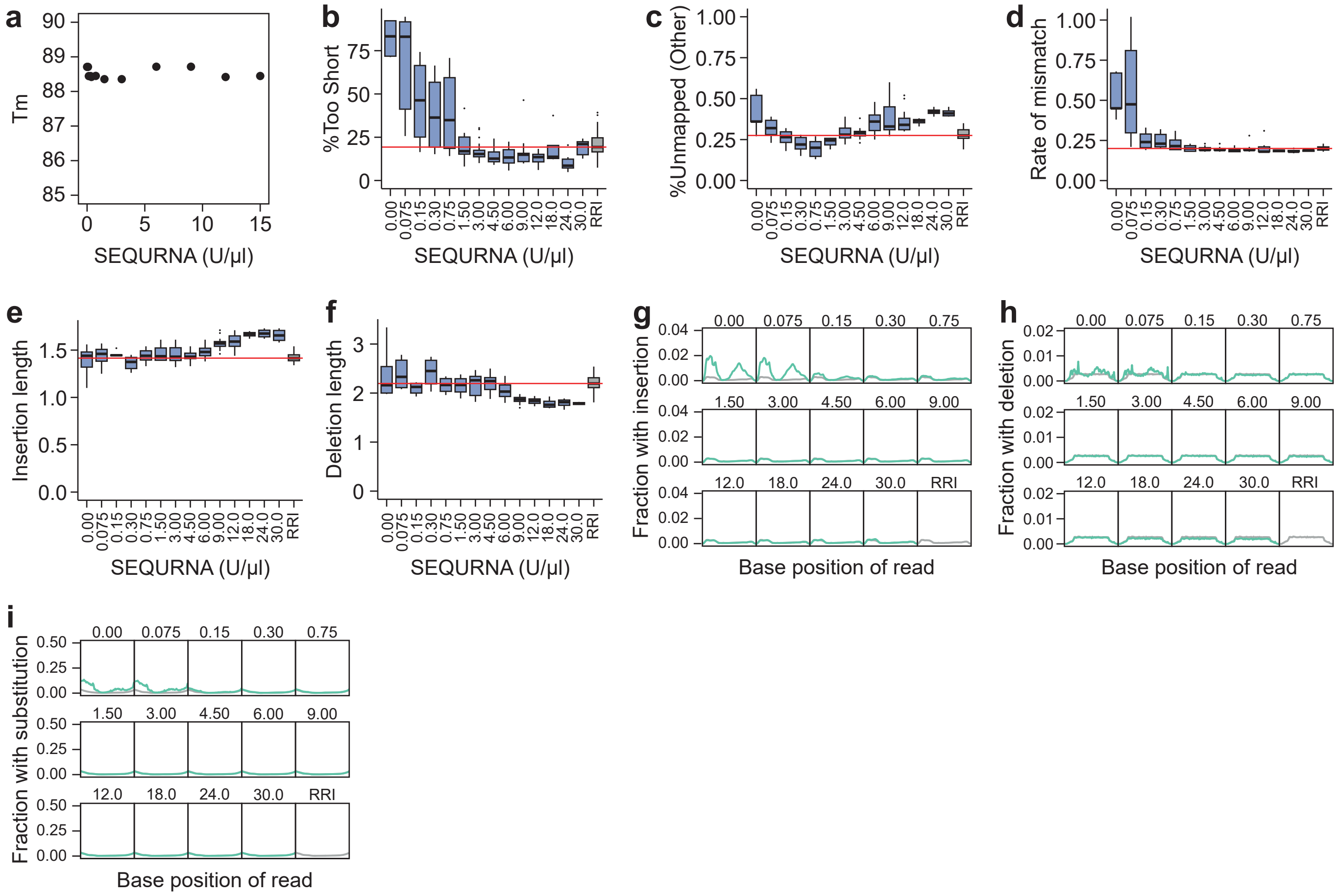

Supplementary Figure 1. Melting temperature and mapping statistics of Smart-seq2 mini-bulk libraries generated using SEQURNA RNase inhibitor.

a. Melting temperatures of a 164 bp DNA duplex under various concentrations of SEQURNA (0-15 U/µl) via denaturation detection using SYBR Green dye fluorescence in a quantitative PCR instrument. **b.** Box plot of percent sequencing reads too short to be mapped to the reference genome for Smart-seq2 (SS2) libraries, generated from 100 pg total mouse RNA and various concentrations of SEQURNA in the SS2 lysis buffer, or standard SS2 lysis buffer using RRI. **c.** Box plot of percent sequencing reads unmapped (other) to the reference genome for SS2 libraries. **d.** Box plot of the mismatch rate in reads of SS2 bulk libraries. **e.** Box plot of the average insertion length of SS2 libraries. **f.** Box plot of the average deletion length in reads of SS2 libraries. **g.** Line plot of fraction of bases along sequencing reads with insertion in SS2 libraries. **h.** Line plot of fraction of bases along sequencing reads with deletion in SS2 libraries. **i.** Line plot of fraction of bases along sequencing reads with substitution in SS2 libraries. **b-i.** n = 5-35 replicates per condition (total = 195).

Supplementary Figure 2

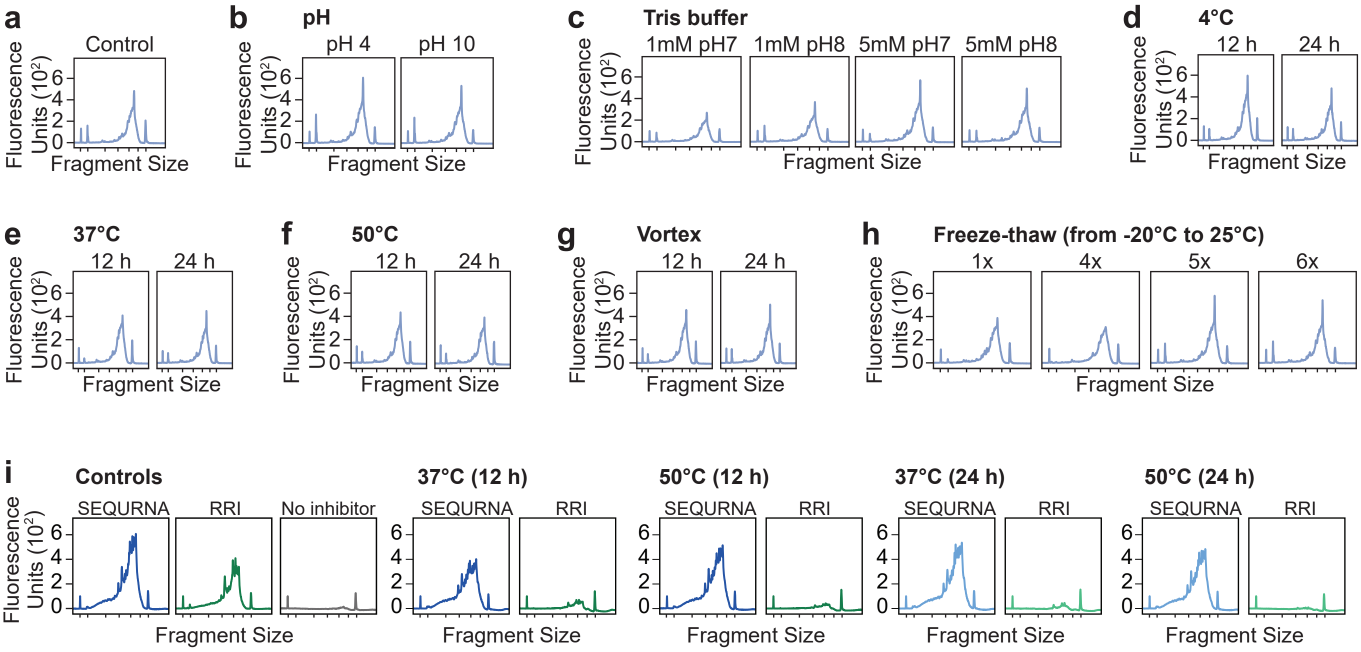

Supplementary Figure 2. Stress test of SEQURNA RNase inhibitor with subsequent Smart-seq2 library generation.

a. Bioanalyzer trace of Smart-seq2 (SS2) cDNA where the lysis buffer contained 3 U/µl SEQURNA (untreated control). **b.** Bioanalyzer traces of SS2 cDNA where the lysis buffer contained 3 U/µl of a SEQURNA which had been subjected to pH 4 (left) or pH 10 (right) using HCl and NaOH respectively before using the inhibitor in lysis buffer. **c.** Bioanalyzer traces of SS2 cDNA where the lysis buffer contained 3 U/µl of a SEQURNA kept in either in a 1mM Tris pH 7 solution, 1mM Tris pH 8 solution, 5mM Tris pH 7 solution, or 5mM Tris pH 8 solution (left to right). **d.** Bioanalyzer traces of SS2 cDNA where the lysis buffer contained 3 U/µl of SEQURNA stock that had been stored at 4°C for 12 hours (left) or 24 hours (right). **e.** Bioanalyzer traces of SS2 cDNA where the lysis buffer contained 3 U/µl of SEQURNA stock that had been stored at 37°C for 12 hours (left) or 24 hours (right). **f.** Bioanalyzer traces of SS2 cDNA where the lysis buffer contained 3 U/µl of SEQURNA stock that had been stored at 50°C for 12 hours (left) or 24 hours (right). **g.** Bioanalyzer traces of SS2 cDNA where the lysis buffer contained 3 U/µl of SEQURNA stock that had been vortexed for 12 hours (left) or 24 hours (right). **h.** Bioanalyzer traces of SS2 cDNA where the lysis buffer contained 3 U/µl of SEQURNA stock that had been freeze-thawed 1, 4, 5, or 6 times before use. Libraries in (**a-h**) were generated from 100 pg MEF total RNA and using 18 PCR cycles in cDNA amplification. **i.** Bioanalyzer traces of SS2 cDNA using 30 pg of mouse liver total RNA as input, where the lysis buffer contained SEQURNA (1.2 U/µl in lysis buffer), RRI (standard SS2), or no RNase inhibitor; and when using RNase inhibitor stocks that had were subjected to heating at 37°C or 50°C for 12 or 24 hours before use in SS2. Liver cDNA amplification was done by 23 PCR cycles. Tick marks on the x-axis correspond to 35, 100, 300, 500, 1000, 3000, and 10380 base pairs.

Supplementary Figure 3

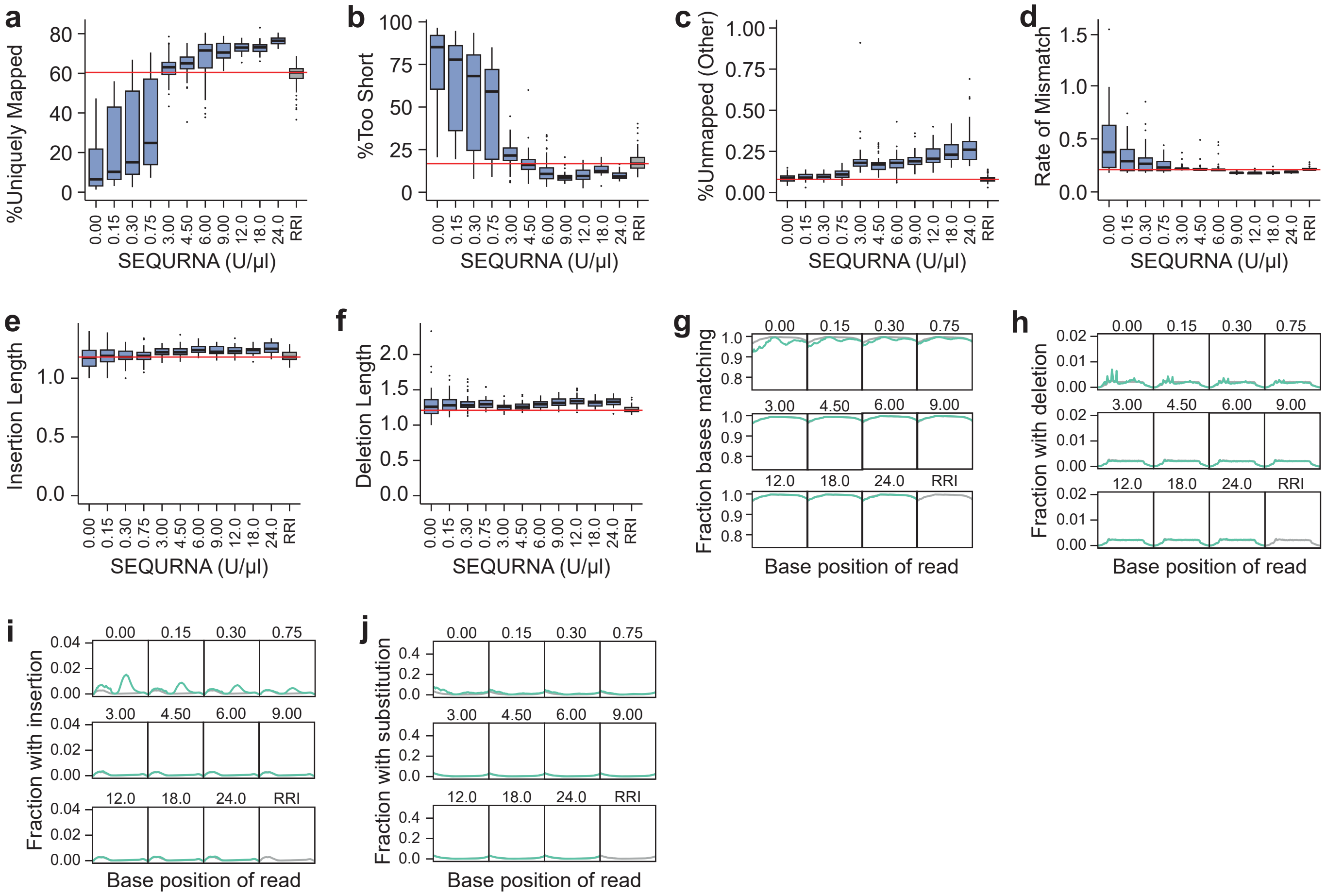

Supplementary Figure 3. Mapping statistics of HEK293FT Smart-seq2 libraries generated using SEQURNA RNase inhibitor.

a. Box plot of percent uniquely mapped sequencing reads to the reference genome for Smart-seq2 (SS2) single-cell libraries generated from HEK293FT cells and various concentrations of SEQURNA in the SS2 lysis buffer or standard SS2 lysis buffer using RRI. n = 37-94 replicates per condition (total= 704). **b.** Box plot of percent sequencing reads too short to be mapped to the reference genome for HEK293FT SS2 libraries. **c.** Box plot of percent sequencing reads unmapped (other) to the reference genome for HEK293FT SS2 libraries. **d.** Box plot of the mismatch rate in HEK293FT SS2 bulk samples. **e.** Box plot of the average insertion length in reads of HEK293FT SS2 libraries. **f.** Box plot of the average deletion length in reads in HEK293FT SS2 libraries. **g.** Line plot of fraction of matching bases along sequencing reads of HEK293FT SS2 libraries. **h.** Line plot of fraction of bases along sequencing reads with deletion in HEK293FT SS2 libraries. **i.** Line plot of fraction of bases along sequencing reads with insertion in HEK293FT SS2 libraries. **j.** Line plot of fraction of bases along sequencing reads with substitution in HEK293FT SS2 libraries.

Supplementary Figure 4

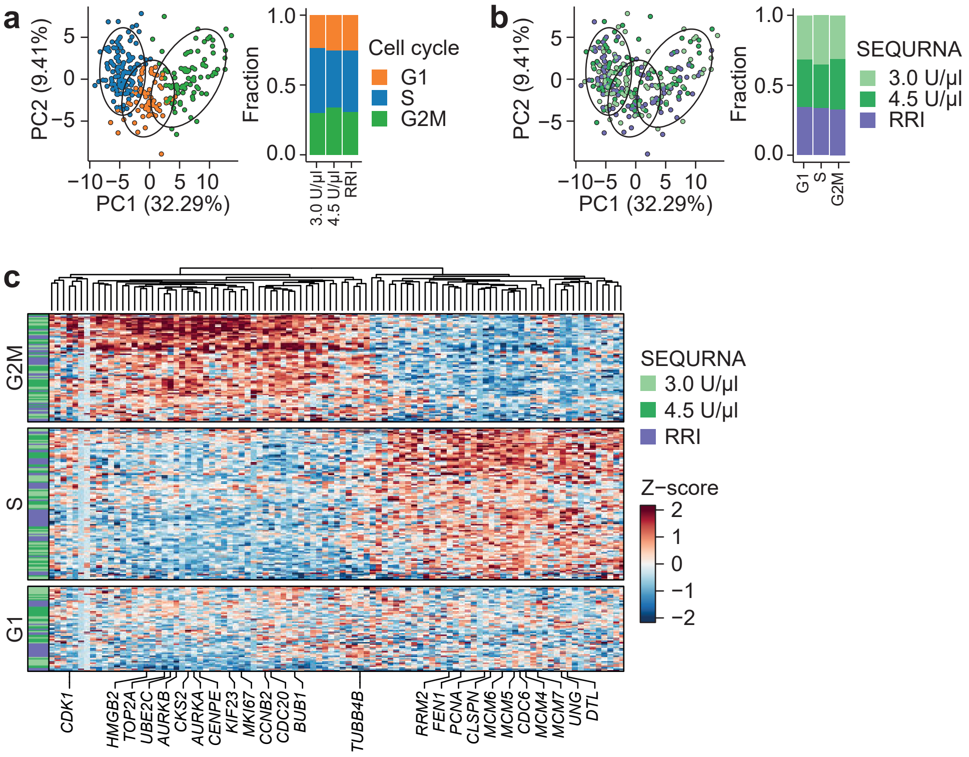

Supplementary Figure 4. Cell cycle analysis of HEK293FT single-cell libraries generated using SEQURNA or recombinant RNase inhibitor.
a. PCA plot for cell-cycle genes for HEK293FT Smart-seq2 (SS2) libraries generated using 3 U/µl (n = 91 cells) and 4.5 U/µl (n = 93 cells) SEQURNA in the lysis buffer, or standard SS2 lysis buffer with recombinant RNase inhibitor (RRI; n = 86 cells), colored according to cell cycle phase. Bar plot of fraction cells in cell cycle phases for each condition is shown to the right. **b.** Same as in (**a**) but coloring PCA cell data points according to RNase inhibitor condition. Bar plot of fraction of cells adhering to each RNase inhibitor condition for each cell cycle phase is show to the right. **c.** Expression-level heatmap of highly variable cell cycle genes grouped by phase for conditions 3 U/µl SEQURNA, 4.5 U/µl SEQURNA, and standard SS2 using RRI.

Supplementary Figure 5

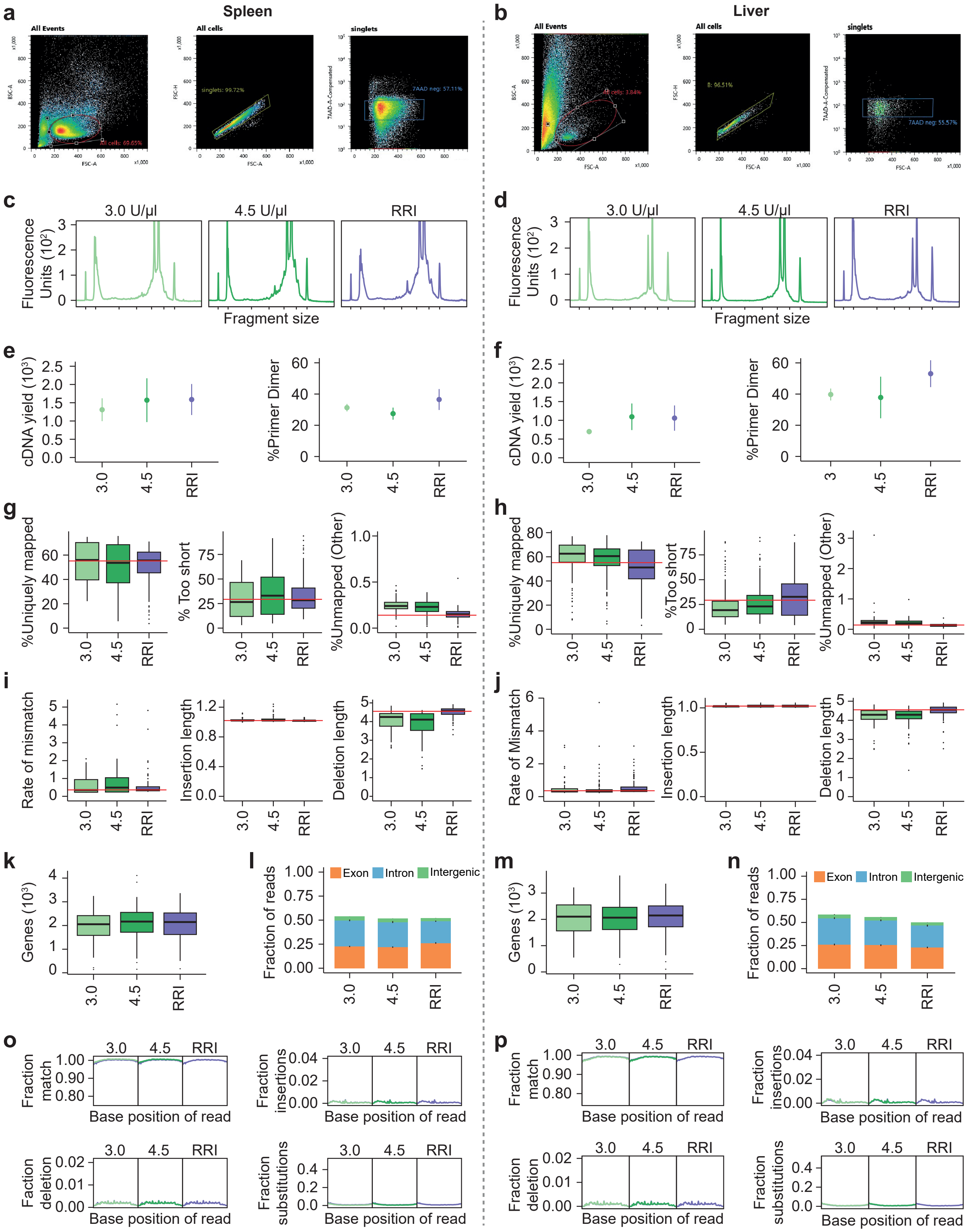

Supplementary Figure 5. FACS gate, cDNA library characteristics, and quality control of liver and spleen cells and scRNAseq libraries.

a. FACS scatter plot of spleen cells gating for lymphocyte population (left), singlets (middle), and viable cells (7AAD-neg) (right). **b.** FACS scatter plot of liver cells gating for lymphocyte population (left), singlets (middle), and viable cells (7AAD-neg) (right). **c.** Bioanalyzer traces of Smart-seq2 (SS2) cDNA libraries generated from sorted spleen cells using 3 and 4.5 U/µl SEQURNA in the lysis buffer, or standard SS2 using recombinant RNase inhibitor (RRI). **d.** Bioanalyzer traces of SS2 cDNA libraries generated from sorted liver cells using 3 and 4.5 U/µl SEQURNA in the lysis buffer, or standard SS2 using RR). Tick marks on the x-axis correspond to 35, 100, 300, 500, 1000, 3000, and 10380 base pairs. **e.** Average cDNA yield (dot) and standard error (whiskers) of SS2 libraries from spleen cells (integration range 200–10,000 bp) (left) and average percent (dot) and range (whiskers) of primer-dimer in SS2 libraries (integration range 20–50 bp) (right). **f.** Same as in (e) but for SS2 libraries from liver. **g.** Box plots of percent uniquely mapped (left), percent sequencing reads too short to be mapped (middle), and percent sequencing reads unmapped (not due to fragments too short or too many mismatches) (right) to the reference genome for spleen cells. **h.** Same as in (g) but for SS2 libraries from liver. **i.** Box plots of rate of mismatch per base (left), average insertion length (middle), and average deletion length (right) for spleen cells. **j.** Same as in (h) but for SS2 libraries from liver. **k.** Box plot of number of genes detected for each RNase inhibitor condition for spleen cells. **l.** Stacked bar plot of fraction reads mapping to exonic, intronic, or intergenic regions of the mouse genome for spleen cells. **m.** Box plot of number of genes detected for each RNase inhibitor condition for liver cells. **n.** Stacked bar plot of fraction reads mapping to exonic, intronic, or intergenic regions of the mouse genome for liver cells. **o.** Line plot of fraction of bases along sequencing reads matching the genome (top left), with an insertion (top right), with a deletion (bottom left), and with a substitution (bottom right) indicating read quality for spleen cells. **p.** Line plot of fraction of bases along sequencing reads matching the genome (top left), with an insertion (top right), with a deletion (bottom left), and with a substitution (bottom right) indicating read quality for liver cells.

Supplementary Figure 6

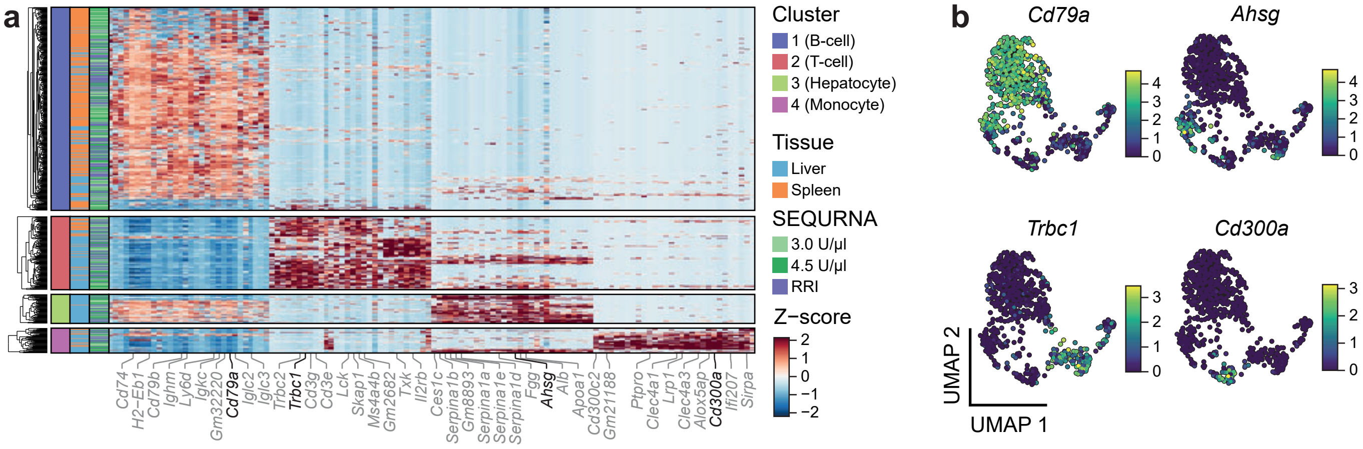

Supplementary Figure 6. Cell type marker gene expression in spleen and liver cells.
a. Expression-level heatmap of top 30 variable genes for each UMAP-generated cluster from single cell RNA sequencing of mouse liver (n = 349) and spleen-derived (n = 368) cells, sorted by gene expression with left bars indicating cell cluster based on transcriptome signature, source tissue, and RNase inhibitor condition (3 or 4.5 U/µl SEQURNA and standard SS2 with recombinant inhibitor), **b.** Mouse liver spleen-cell UMAP coloured by expression level of selected variable genes for each cell clusters: *Cd79a*, *Trbc1*, *Ahsg*, and *Cd300a*.

Supplementary Figure 7

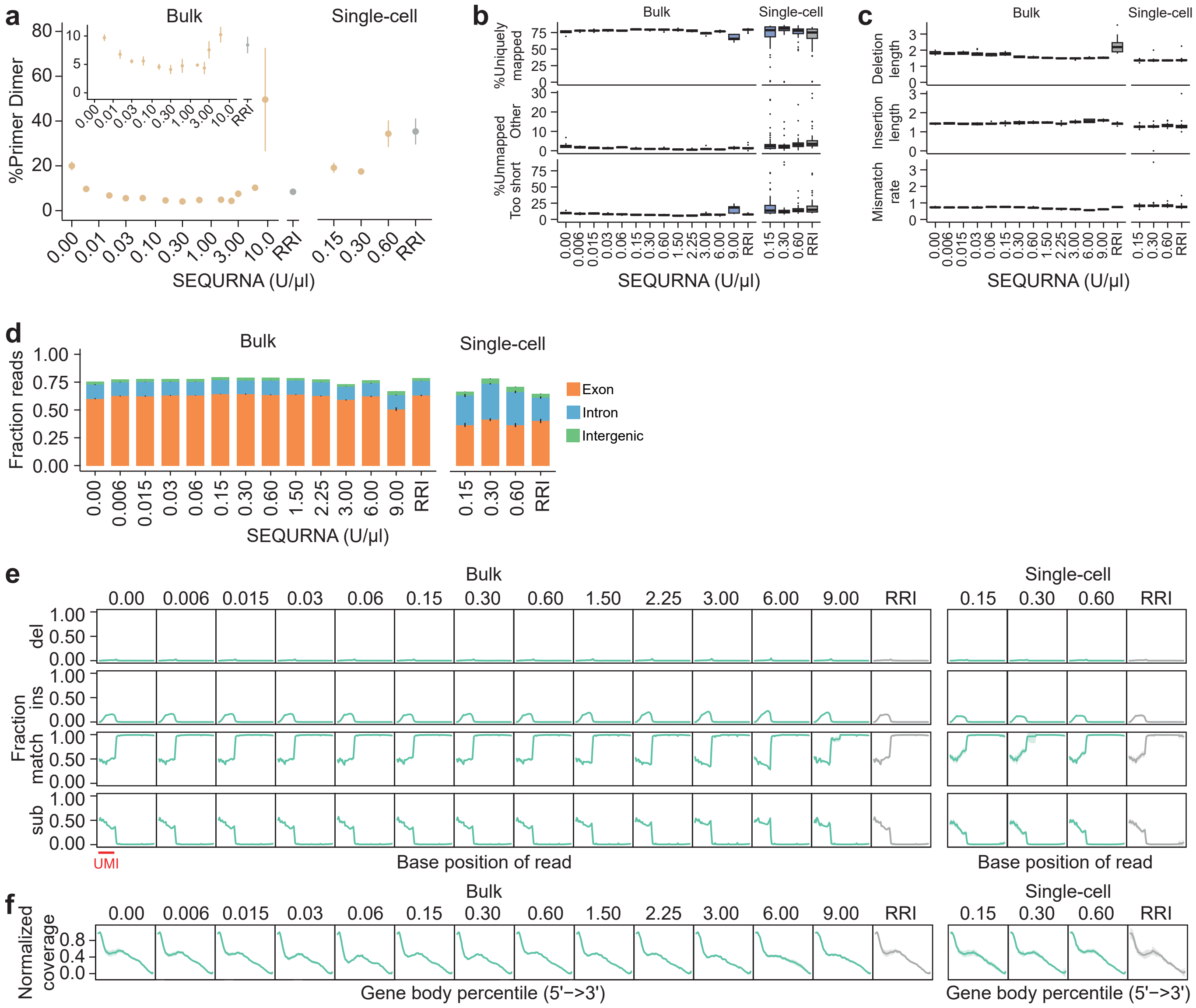

Supplementary 7. Mapping statistics of Smart-seq3 libraries generated using SEQURNA RNase inhibitor. a. Average percent (dot) and range (whiskers) of primer-dimer in Smart-seq3 (SS3) cDNA libraries from 100 pg of mouse total RNA and individual HEK293FT cells (integration range 20–50 bp). The inset corresponds to a zoom of the figure from 0–15%, highlighting the trend of decreasing primer-dimer occurrence with increasing concentration of SEQURNA up to the higher end of the “optimal range”. **b.** Box plots of percent uniquely mapped to the reference genome (top), percent sequencing reads too short to be mapped (middle), and percent sequencing reads unmapped (not due to fragments too short or too many mismatches) (bottom) for 100 pg mouse total RNA and individual HEK293FT cells. **c.** Box plot of average insertion length (top), average deletion length (middle), and rate of mismatch per base (bottom) for 100 pg mouse total RNA and individual HEK293FT cells. **d.** Stacked bar plot of fraction reads mapping to exonic, intronic, or intergenic regions of the genome for 100 pg mouse total RNA and individual HEK293FT cells. **e.** Line plot of fraction of bases along sequencing reads with a deletion, with an insertion, matching the genome, and with a substitution indicating read quality for libraries from 100 pg mouse RNA and individual HEK293FT cells. **f.** Normalized gene body coverage of mapped reads along transcripts for libraries from 100 pg mouse total RNA and individual HEK293FT cells.

Supplementary Figure 8

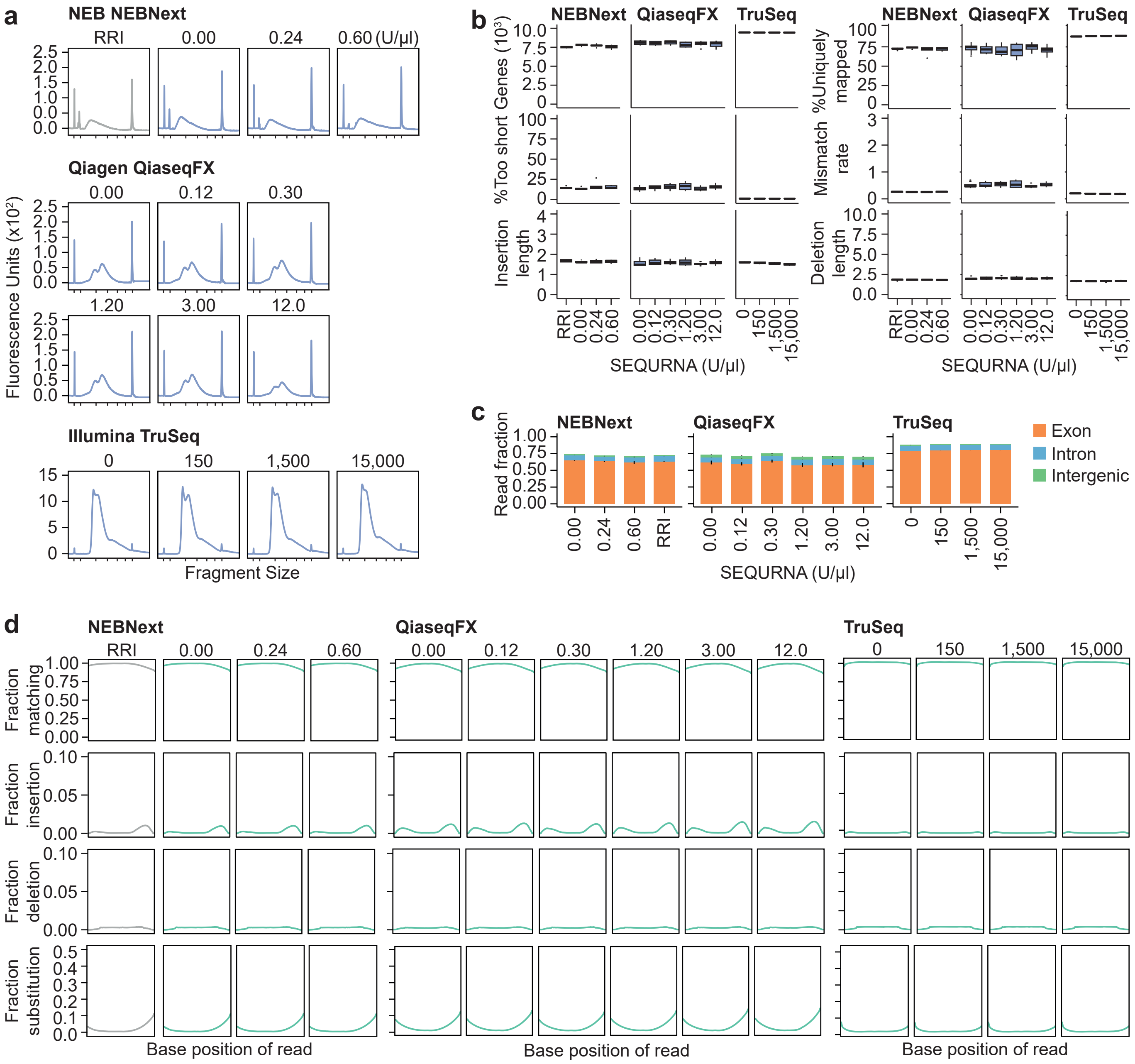

Supplementary Figure 8. SEQURNA compatibility with commercial RNA-seq kits.
a. Bioanalyzer plots of final RNA sequencing libraries generated from three different sequencing kits. Top: NEBNext RNA Sequencing Kit with 100 pg of mouse total RNA and varying amounts of SEQURNA (0, 0.24, or 0.6 U/µL) or RRI added in the lysis step. Middle: Bioanalyzer trances of sequencing libraries generated from the QIAseq FX Single Cell RNA Library Kit with 100 pg of mouse total RNA and varying amounts of SEQURNA (0, 0.12, 0.3, 1.2, 3, or 12 U/µl) added to the denaturation step. Bottom: Bioanalyzer plots of RNA-sequencing libraries generated from the TruSeq RNA Sample Preparation Kit with 100 ng of mouse total RNA in a storage buffer containing varying amounts of SEQURNA (0, 150, 1,500, or 15,000 U/µl) prior to adding the RNA sample to the RNA purification oligo-dT beads. With this protocol, including a bead capture step of polyadenylated RNA, high SEQURNA concentrations were considered, evaluating whether detrimental carryover to the following library preparation protocol occurred. Tick marks on the x-axis correspond to 35, 100, 300, 500, 1000, 3000, and 10380 base pairs. **b.** Box plot of number of genes detected for each inhibitor condition used to each kit (top left), box plot of percent uniquely mapped sequencing reads to the reference genome for each inhibitor condition for the kits (top right), box plot of percent sequencing reads too short to be mapped to the reference genome for kit samples (middle left), box plot of the mismatch rate length for kit samples (middle right), box plot of the average insertion length for kit samples (bottom left), and box plot of the average deletion length for kit samples (bottom right). **c.** Stacked bar plot of fraction reads mapping to exonic, intronic, or intergenic regions of the mouse genome. **d.** From top to bottom: line plot of fraction of bases along sequencing reads matching the genome, indicating sequencing read quality, line plot of fraction of bases along sequencing reads with insertion for kit samples, line plot of fraction of bases along sequencing reads with deletion for kit samples, and line plot of fraction of bases along sequencing reads with substitution for kit samples.

Supplementary Figure 9

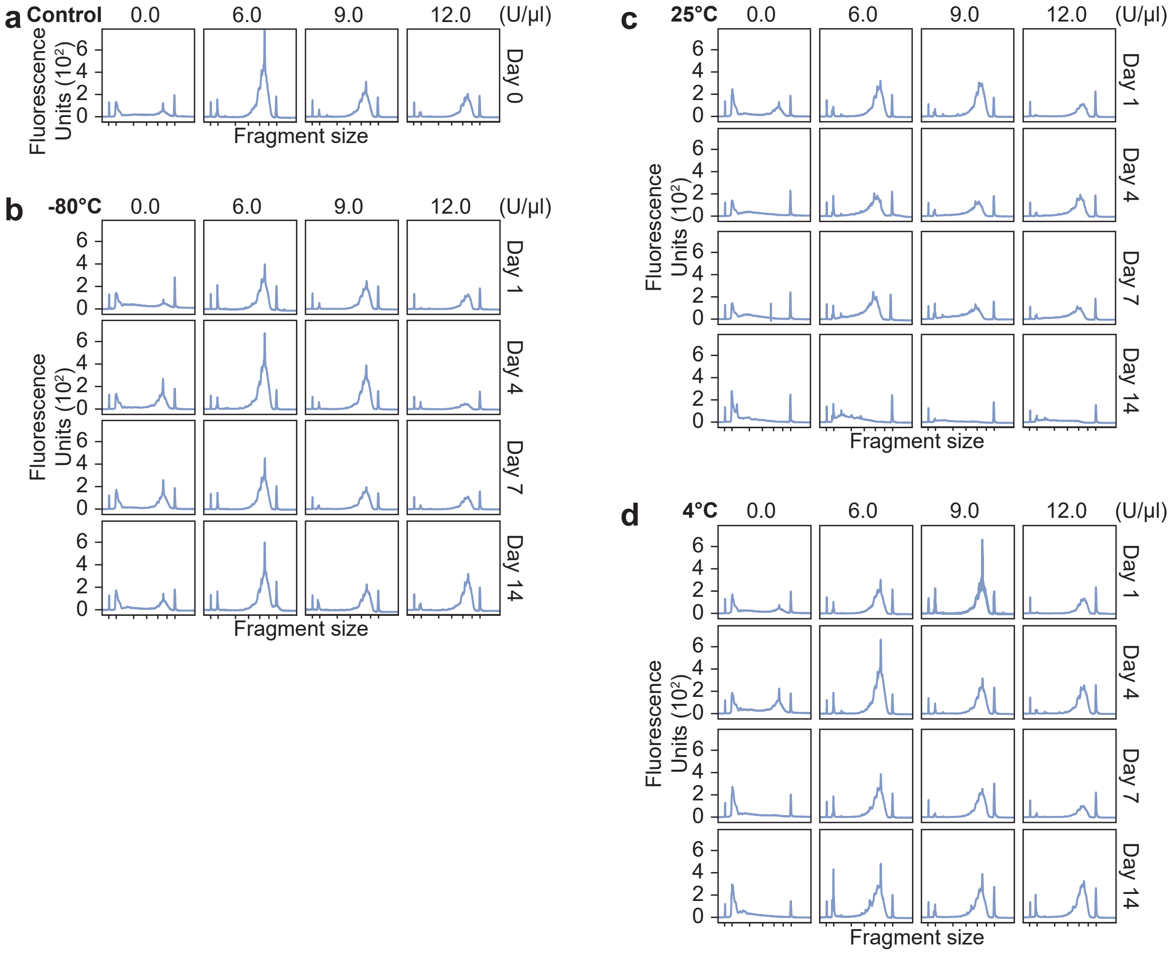

Supplementary Figure 9. SEQURNA delays RNA degradation of cells stored long-term in Smart-seq2 lysis buffer.
a. Bioanalyzer traces of control Smart-seq2 (SS2) cDNA libraries from HEK293FT cells FACS-sorted into lysis buffer containing either no inhibitor, 6, 9 or 12 U/µl of SEQURNA, where cDNA was generated immediately after cell sorting (day 0). **b.** Bioanalyzer traces of SS2 cDNA libraries from HEK293FT cells FACS-sorted into lysis buffer containing various concentrations of inhibitor processed into libraries after storage at -80°C for 1, 4, 7, or 14 days (top to bottom). **c.** Bioanalyzer traces of SS2 cDNA libraries from HEK293FT cells FACS-sorted into lysis buffer containing various concentrations of inhibitor after storage at 25°C for 1, 4, 7, and 14 days (top to bottom). **d.** Bioanalyzer traces of SS2 cDNA libraries from HEK293FT cells FACS-sorted into lysis buffer containing various concentrations of inhibitor after storage at 4°C for 1, 4, 7, and 14 days (top to bottom). Tick marks on the x-axis correspond to 35, 100, 300, 500, 1000, 3000, and 10380 base pairs.

Supplementary Note 1:
Smart-seq2 with SEQURNA thermostable RNase inhibitor

In this version of the Smart-seq2 protocol, the use of a recombinant RNase inhibitor is replaced by the SEQURNA synthetic thermostable RNase inhibitor (RI). The overall protocol differs from the standard Smart-seq2 protocol only in that the SEQURNA RI is added only to the cell lysis buffer (i.e., cell collection buffer) and **not** supplemented again in the Reverse Transcription step. This is because the SEQURNA RI remains effective throughout the cell lysis and RNA denaturation step at 72°C and the following Reverse Transcription, while the protein-based Recombinant RNase inhibitor used in the original Smart-seq2 protocol, may denature and lose RI capacity during heating.

Important notes:
-Note that the optimal SEQURNA RI concentration in Smart-seq3 and is different than for Smart-seq2.

-Note that using more RNase inhibitor than indicated amounts is not beneficial. Excessive amounts of added inhibitor may result in decreased cDNA library yield and quality.

-Slight variations or modifications of the Smart-seq2 protocol are used in different labs, e.g., slight change in lysis buffer detergent or concentration: Use the suggested concentration of SEQURNA RI in the Smart-seq2 lysis buffer independent of Smartseq2 version.

-For a detailed protocol description and the subsequent sequencing library generation steps and indexing, refer to the original Smart-seq2 protocol:
Picelli 2014, Nature Protocols
https://www.nature.com/articles/nprot.2014.006

-For further details on the development of Smart-seq2, refer to the original Smartseq2 paper:
Picelli 2013, Nature Methods
https://www.nature.com/articles/nmeth.2639

-For in-depth information about the SEQURNA RNase inhibitor as well as potential updates of the protocol, please visit www.sequrna.com.

Abbreviations
DTT – Dithiothreitol
RI – RNase inhibitor
RT – Reverse Transcription
SS2 – Smart-seq2
TSO – Template-Switching Oligo

Oligonucleotide sequences (5’ to 3’):
SS2 oligo dT: 5′–AAGCAGTGGTATCAACGCAGAGTACT30VN-3′

SS2 TSO: 5′-AAGCAGTGGTATCAACGCAGAGTACATrGrG+G-3′
ISPCR: 5′-AAGCAGTGGTATCAACGCAGAGT-3′

Prepare lysis plates

Prepare lysis buffer mix:

Note: Optimal concentration range of SEQURNA RI in the Smart-seq2 protocol is between 1-2 Mass U/µL in the lysis buffer, resulting in 0.45-0.9 Mass U/µL in the following RT step.

Reagent	Conc. in lysis buffer	µL per reaction	96 well plate (110 rxns)	384 well plate (410 rxns)
0.2% Triton X-100	0.08%	1.9	209	779
SEQURNA RI (50 Mass U/µL)	1.2 Mass U/µL	0.11	11	41
dNTPs mix (10 mM)	2.2 mM	1	110	410
SS2 oligo dT primer (10 µM)	2.2 µM	1	110	410
Nuclease-free water	-	0.49	53.9	200.9
ERCC spike-ins (Optional)	-	-	-	-
Total		4.5 µL	495 µL	1845 µL

Add 4.5 µL lysis buffer to each well of a 96/384 well plate, and centrifuge briefly to collect lysis buffer in the bottom of the wells.

Sample collection

Sort single cells into 4.5 µL of lysis buffer lysis in either 96 or 384 wells.

Seal the plate with appropriate cover seals (tolerating -80°C to >100°C) and centrifuge the finished sorted plate immediately after. Transfer the plate to a -80°C freezer if not processing the cells into cDNA libraries within 1 day (keep plates in ~4°C fridge up to 1 day).

Cell lysis

Remove the plate of sorted cells from the -80°C freezer and incubate in a thermocycler with heated lid at 72 °C for 3 min, followed by a 4 °C hold. Ensure that the plate is properly sealed, to avoid evaporation (use thermal pads, depending on thermocycler model).

Reverse Transcription

While the plate is incubating at the cell lysis step, prepare the following Reverse transcription master-mix.

Note: Do **not** add additional inhibitor in the reverse transcription step. The SEQURNA RI from the lysis buffer stays effective throughout lysis and the following RT.

Reagent	Reaction conc.	µL per reaction	96 well plate (110 rxns)	384 well plate (410 rxns)
SuperScript II reverse transcriptase (200 U/µL)	100 U	0.5	55	205
Superscript II First Strand buffer (5x)	1x	2	220	820
DTT (100 mM)	5 mM	0.5	55	205
Betaine (5 M)	1 M	2	220	820
$MgCl_2$ (1 M)	10 mM	0.1	11	41
TSO (100 µM)	1 µM	0.1	11	41
Nuclease-free water		0.3	33	123
Total		5.5 µL	605 µL	2255 µL

Add 5.5 µL RT mix to each well of a 96/384 well plate.

Replace the storage seal with a PCR seal. Ensure that the plate is properly sealed to avoid evaporation (use thermal pads, depending on thermocycler model).

Briefly centrifuge to collect reaction at the bottom of the tube.

Incubate the plate in a thermocycler at:

Temp	Time	Cycles
42 °C	90 min	1x
50 °C	2 min	10x
42 °C	2 min	
72 °C	15 min	1x
4 °C	Hold	Hold

Preamplification PCR

Start preparing the PCR mix, when the incubation of the reverse transcription reaction is near completion, by combining the following components.

Reagent	Reaction conc.	µL per reaction	96 well plate (110 rxns)	384 well plate (410 rxns)
First-strand reaction	–	10	1100	4100
KAPA HiFi HotStart ReadyMix (2x)	1x	12.5	1375	5125
ISPCR primers (10 µM)	0.08 µM	0.2	22	82
Nuclease-free water	–	2.3	253	943
Total volume	–	25 µL	2750 µL	10250 µL

Add 15 µL PCR mix to each well of a 96/384 well plate.

Briefly centrifuge to collect reaction at the bottom of the plate. Seal with a new PCR seal. Ensure that the plate is properly sealed, to avoid evaporation (use thermal pads, depending on thermocycler model).

Incubate the plate in a thermocycler at:

Step	Temp	Time	Cycles
Initial denaturation	98 °C	3 min	1x
Denaturation	98 °C	20 sec	18-25x*
Annealing	67°C	15 sec	
Elongation	72 °C	6 min	
Final Elongation	72 °C	5 min	1x
Hold	4 °C	Hold	

* depending on cell type (reflecting RNA content per cell)

# cDNA purification

Purification of cDNA is performed using Ampure XP beads or equivalent, e.g., 22% PEG Clean-up Beads (https://www.protocols.io/view/smart-seq3-protocol36wgq5rjxgk5).

1. To purify cDNA, add 0.8:1 ratio of beads to sample (20 µL) and mix by gently pipetting up and down.

2. Incubate at room temperature for 8 min.

3. Place on magnet and allow beads to settle ~5 min.

4. Discard supernatant, and wash pellets with freshly prepared 80% ethanol, keeping the plate on the magnet. The volume of ethanol will depend on the type of plate used (e.g. ~100 µL in case of 96-well plate).

5. Remove ethanol and repeat step 4.

6. Remove all ethanol and let the beads air dry for 2-5 min (do not over-dry the pellets).

7. Elute cDNA by adding 18 µL UltraPure Water or other suitable elution buffer (e.g., 10mM Tris-HCl, pH8.5) onto the pellets.

8. Remove the plate from the magnet and resuspend beads by pipetting up and down. Incubate for 8 min.

9. Place on magnet until clear (~3 min) and collect the eluate, containing the purified cDNA, to fresh plates or tubes.

Quality Control check

Inspect the cDNA library concentration and size distribution, e.g., on an Agilent Bioanalzyer High Sensitivity DNA Analysis chip.

Representative Bioanalyzer image of successfully amplified Smart-seq2 cDNA from a HEK cell using the SEQURNA RI:

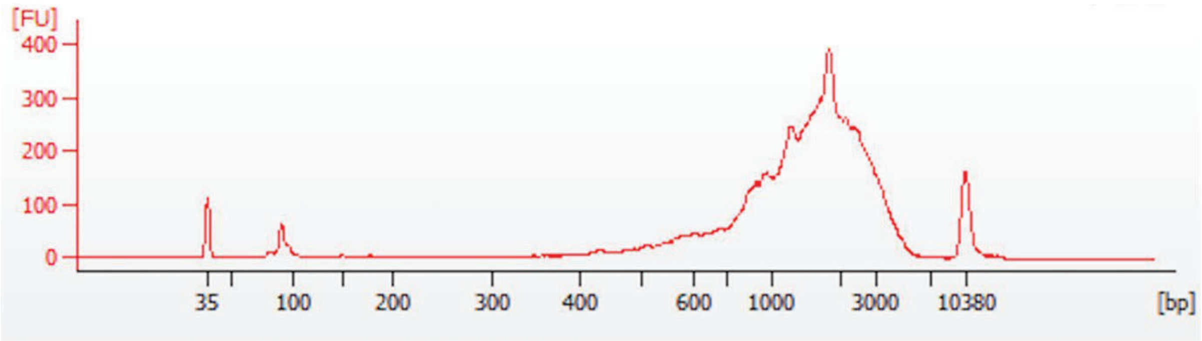

Trace of Smart-seq2 cDNA trace from a HEK cell, using an Agilent Bioanalzyer High Sensitivity DNA Analysis chip.

To prepare indexed sequencing libraries from Smart-seq2 cDNA by tagmentation and PCR, please refer to Picelli 2014, Nature Protocols: https://www.nature.com/articles/nprot.2014.006

Supplementary Note 2
Smart-seq3 with SEQURNA thermostable RNase inhibitor

In this version of the Smart-seq3 protocol, the use of a recombinant RNase inhibitor is replaced by the SEQURNA synthetic thermostable RNase inhibitor (RI). The overall protocol differs from the standard Smart-seq3 protocol only in that the SEQURNA RI is added only to the cell lysis buffer (i.e., cell collection buffer) and **not** supplemented again in the Reverse Transcription step. This is because the SEQURNA RI remains effective throughout the cell lysis and RNA denaturation step at 72°C and the following Reverse Transcription, while the protein-based Recombinant RNase inhibitor used in the original Smart-seq3 protocol, may denature and lose RI capacity during heating.

Important notes:

-Note that the optimal SEQURNA RI concentration in Smart-seq2 lysis buffer is different than for Smart-seq3.

-Note that using more RNase inhibitor than indicated amounts is not beneficial. Excessive amounts of added inhibitor may result in decreased cDNA library yield and quality.

-Slight variations or modifications of the Smart-seq3 protocol are used in different labs, e.g., slight change in lysis buffer detergent or concentration: Use the suggested concentration of SEQURNA RI in the Smart-seq3 lysis buffer independent of Smartseq3 version.

-For a detailed protocol description and the subsequent sequencing library generation steps and indexing, refer to the online protocol on protocols.io:
https://dx.doi.org/10.17504/protocols.io.bcq4ivyw

-For further details on the development of Smart-seq3, refer to the original Smartseq3 paper:
Hagemann-Jensen 2020, Nature Biotechnology
https://www.nature.com/articles/s41587-020-0497-0

-For in-depth information about the SEQURNA RNase inhibitor as well as potential updates of the protocol, please visit www.sequrna.com.

Abbreviations

DTT – Dithiothreitol
RI – RNase inhibitor
RT – Reverse Transcription
SS3 – Smart-seq3
TSO – Template-Switching Oligo

Oligonucleotide sequences (5’ to 3’):
SS3 oligo dT: 5′-/5Biosg/ACGAGCATCAGCAGCATACGAT30VN-3′

SS3 TSO: 5′-/5Biosg/AGAGACAGATTGCGCAATGNNNNNNNNrGrGrG-3′
SS3 Fwd Primer:
5′-TCGTCGGCAGCGTCAGATGTGTATAAGAGACAGATTGCGCAA*T*G-3′
SS3 Rev Primer: 5′-ACGAGCATCAGCAGCATAC*G*A-3′
* phosphorothioate bonds

Prepare lysis plates

Prepare lysis buffer mix:
Note: Optimal concentration of SEQURNA RI in the Smart-seq3 protocol is 0.15-0.3 Mass U/μL in the lysis buffer, resulting in 0.11-0.23 Mass U/μL in the following RT step.

Reagent	Conc. in lysis buffer	μL per reaction	96 well plate (110 rxns)	384 well plate (410 rxns)
Poly-ethylene Glycol 8000 (50% solution)	6.7%	0.40	44	164
Triton X-100 (10% solution)	0.1%	0.03	3.3	12.3
SEQURNA Inhibitor (50 Mass U/μL)	0.2 Mass U/μL	0.012	1.32	4.92
SS3 oligo dT (100μM)	0.67μM	0.02	2.2	8.2
dNTPs (25mM/each)	0.67 mM/each	0.08	8.8	32.8
Nuclease Free Water	-	2.46	270.6	1008.6
ERCC spike-ins (Optional)	-	-	-	-
Total	-	3 μL	330 μL	1230 μL

Add 3 μL lysis buffer to each well of a 96/384 well plate, and centrifuge briefly to collect lysis buffer in the bottom of the wells.

Sample collection

Sort single cells into 3 μL of lysis buffer lysis in either 96 or 384 wells.

Seal with appropriate seals (tolerating -80°C to >100°C) and centrifuge the finished sorted plate immediately after. Transfer the plate to a -80°C freezer if not processing the cells into cDNA libraries within 1 day (keep plates in ~4°C fridge up to 1 day).

Cell lysis

Remove the plate of sorted cells from the -80°C freezer and incubate in a thermocycler with heated lid at 72 °C for 3 min, followed by a 4 °C hold. Ensure that the plate is properly sealed, to avoid evaporation (use thermal pads, depending on thermocycler model).

Reverse Transcription

While the plate is incubating at the cell lysis step, prepare the following Reverse transcription master-mix.

Note: Do **not** add additional inhibitor in the reverse transcription step. The SEQURNA RI from the lysis buffer stays effective throughout lysis and the following RT.

Reagent	Conc. in RT	µL per reaction	96 well plate (110 rxns)	384 well plate (410 rxns)
Tris-HCl pH 8.3 (1M)	25mM	0.1	11	41
NaCl (1M)	30mM	0.12	13.2	49.2
$MgCl_2$ (100mM)	2.5mM	0.1	11	41
GTP (100mM)	1mM	0.04	4.4	16.4
DTT (100mM)	8mM	0.32	35.2	131.2
SS3 TSO (100µM)	2µM	0.08	8.8	32.8
Maxima H-minus RT enzyme (200U/µL)	8U	0.04	4.4	16.4
Nuclease Free Water	-	0.2	22	82
Total				
	-	1µL	110µL	410µL

Add 1 µL RT mix to each well of a 96/384 well plate.

Replace the storage seal with a PCR seal. Ensure that the plate is properly sealed to avoid evaporation (use thermal pads, depending on thermocycler model).

Briefly centrifuge to collect reaction at the bottom.

Incubate the plate in a thermocycler at:

Temp	Time	Cycles
42 °C	90 min	1x
50 °C	2 min	10x
42 °C	2 min	
85 °C	5 min	1x
4 °C	Hold	Hold

Preamplification PCR

Start preparing the PCR mix, when the incubation of the reverse transcription reaction is near completion, by combining the following components.

Reagent	Conc. in PCR	µL per reaction	96 well plate (110 rxns)	384 well plate (410 rxns)
Kapa HiFi HotStart buffer (5X)	1X	2.0	220	820
dNTPs (25mM/each)	0.3mM/each	0.12	13.2	49.2
$MgCl_2$ (100mM)	0.5mM	0.05	5.5	20.5
Fwd Primer (100µM)	0.5µM	0.05	5.5	20.5
Rev Primer (100µM)	0.1µM	0.01	1.1	4.1
Kapa Polymerase (1U/µL)	0.02U/µL	0.2	22	82
Nuclease Free Water	–	3.57	392.7	1463.7
Total	–	6µL	660µL	2460µL

Add 6 µL PCR mix to each well of a 96/384 well plate.

Briefly centrifuge to collect reaction at the bottom. Seal with a new PCR seal. Ensure that the plate is properly sealed to avoid evaporation (use thermal pads, depending on thermocycler model).

Incubate the plate in a thermocycler at:

Step	Temp	Time	Cycles
Initial denaturation	98 °C	3 min	1x
Denaturation	98 °C	20 sec	18-25x*
Annealing	65°C	30 sec	
Elongation	72 °C	4 min	
Final Elongation	72 °C	5 min	1x
Hold	4 °C	Hold	

* depending on cell type (reflecting RNA content per cell)

# cDNA purification

Purification of cDNA is performed using Ampure XP beads or equivalent, e.g., 22% PEG Clean-up Beads (https://www.protocols.io/view/smart-seq3-protocol36wgq5rjxgk5).

1. To purify cDNA add 0.8:1 ratio of 22% PEG beads to sample, and mix by gently pipetting up and down.

2. Incubate at room temperature for 8 min.

3. Place on magnet and allow beads to settle for ~5 min.

4. Discard supernatant, and wash once with 20 µL/ 100 µL of freshly prepared 80% ethanol for 384 / 96 well plates respectively, keeping the plate on the magnet.

5. Remove ethanol and let the beads air dry for 2-5 min (do not over-dry the pellets).

6. Elute cDNA in 12 µL of UltraPure Water or other suitable elution buffer (e.g., 10mM Tris-HCl, pH8.5) onto the pellets.

7. Remove the plate from the magnet and resuspend beads by pipetting up and down. Incubate for 8 min.

8. Place on magnet until clear (~3 min) and collect the eluate, containing the purified cDNA, to fresh plates or tubes.

<u>Quality Control check</u>
Inspect the cDNA library concentration and size distribution, e.g., on an Agilent Bioanalzyer High Sensitivity DNA Analysis chip.

Representative Bioanalyzer image of successfully amplified Smart-seq3 cDNA from a HEK cell using the SEQURNA RI:

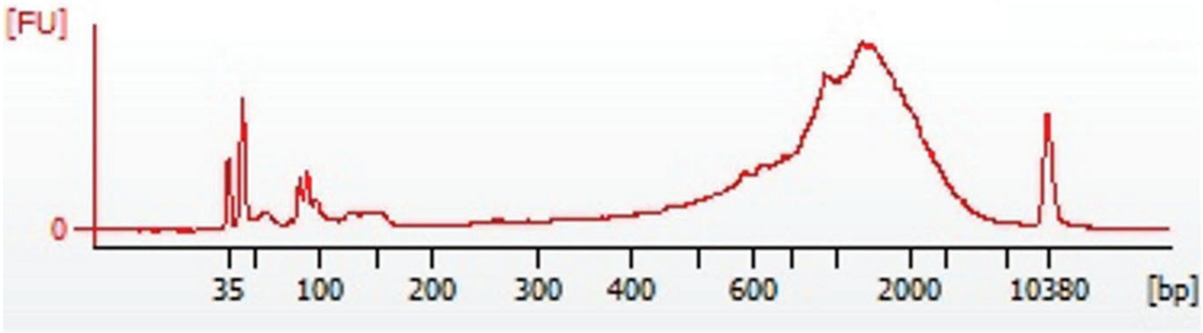

Trace of Smart-seq3 cDNAfrom a HEK cell, using an Agilent Bioanalzyer High Sensitivity DNA Analysis chip.

To prepare indexed sequencing libraries from Smart-seq3 cDNA by tagmentation and PCR, please refer to online protocol https://dx.doi.org/10.17504/protocols.io.bcq4ivyw.

Supplementary Note 3:
Smart-seq3xpress with SEQURNA thermostable RNase inhibitor

In this version of the Smart-seq3xpress protocol, the use of a recombinant RNase inhibitor is replaced by the SEQURNA synthetic thermostable RNase inhibitor (RI). The overall protocol differs from the standard Smart-seq3xpress protocol only in that the SEQURNA RI is added only to the cell lysis buffer (i.e., cell collection buffer) and **not** supplemented again in the Reverse Transcription step. This is because the SEQURNA RI remains effective throughout the cell lysis and RNA denaturation step at 72°C and the following Reverse Transcription, while the protein-based Recombinant RNase inhibitor used in the original Smart-seq3xpress protocol, may denature and lose RI capacity during heating.

Important notes:
-Note that using more RNase inhibitor than indicated amounts is not beneficial. Excessive amounts of added inhibitor may result in decreased library yield and quality.

-This protocol requires a liquid handler capable of nanoliter dispenses.

- For a detailed protocol description and the subsequent sequencing library generation steps, refer to the online protocol on protocols.io:
https://www.protocols.io/view/smart-seq3xpress-yxmvmk1yng3p/v2

-For further details on the development of Smart-seq3xpress, refer to the original Smart-seq3xpress paper:
Hagemann-Jensen 2022, Nature Biotechnology
https://www.nature.com/articles/s41587-022-01311-4

-For in-depth information about the SEQURNA RNase inhibitor, please refer to our white paper available at www.sequrna.com.

Abbreviations
DTT – Dithiothreitol
RI – RNase inhibitor
RT – Reverse Transcription
TSO – Template-Switching Oligo

Oligonucleotide sequences (5' to 3'):
SS3 oligo dT: 5′-/5Biosg/ACGAGCATCAGCAGCATACGAT30VN-3′
Smart-seq3xpress TSO: 5′-/5BiosG/AGAGACAGATTGCGCAATGNNNNNNNNWWrGrGrG-3′
SS3 Fwd Primer: 5′-TCGTCGGCAGCGTCAGATGTGTATAAGAGACAGATTGCGCAA*T*G-3′
SS3 Rev Primer: 5′-ACGAGCATCAGCAGCATAC*G*A-3′
* phosphorothioate bonds

Prepare overlay plates

Use Vapor-Lock, Sillicone oil 25 cSt, Silicone Oil 100 cSt (The higher viscosity is better suited for shipping plates). CAUTION: Do not dispense these silicone oils / overlays with your non contact liquid handler. The solutions can "creep" everywhere. Use either manual multichannel pipettes or semi-manual (e.g. Integra ViaFlow) / automatic dispensing (e.g. Agilent Bravo, Tecan Fluent) with tips, and prepare and store in bulk.

Add 3 µL of overlay to each well of a 384 well plate. The amount of overlay can be increased if desired.

Quick pulse centrifugation to 1000 x g to ensure all is collected in the bottom of wells.

Put on seal and store at Room temperature until use.

Prepare lysis plates

Prepare lysis buffer mix:
Note: Optimal concentration of SEQURNA RI in the Smart-seq3xpress lysis buffer is 0.2 Mass U/µL, resulting in 1.5 Mass U/µL in the following RT step.

Reagent	Conc. in lysis buffer	µL per reaction	384 well plate (500 rxns)
Poly-ethylene Glycol 8000 (40% solution)	6.7%	0.05	25
Triton X-100 (10% solution)	0.1%	0.003	1.5
SEQURNA Inhibitor (50 Mass U/µL)	0.2 Mass U/µL	0.0012	0.6
SS3 oligo dT (10µM)	0.167µM	0.005	2.5
dNTPs (10mM/each)	0.66mM/each	0.02	10
Nuclease Free Water	-	0.221	110.5
ERCC spike-ins (Optional)	-	-	-
Total	-	0.3 µL	150 µL

Add 0.3 µL lysis buffer to each well of a 384 well plate containing overlay, and centrifuge briefly to collect lysis buffer.

Sample collection

Sort single cells into 0.3 µL of lysis buffer with overlay in 384 wells.

Seal with appropriate seals (tolerating -80°C to >100°C) and centrifuge the finished sorted plate immediately. Transfer the plate to a -80°C freezer if not processing the cells into cDNA libraries within 1 day (or keep plates in ~4°C fridge up to 1 day).

Cell lysis

Remove the plate of sorted cells from the -80°C freezer and incubate in a thermocycler with heated lid at 72 °C for 10 min, followed by a 4 °C hold.

Reverse Transcription

While the plate is incubating at the cell lysis step, prepare the following Reverse transcription master-mix.

Note: Do **not** add additional inhibitor in the reverse transcription step. The SEQURNA RI from the lysis buffer stays effective throughout lysis and the following RT.

Reagent	Conc. in RT	µL per reaction	384 well plate (500 rxns)
Tris-HCl pH 8.3 (1M)	25mM	0.01	5
NaCl (2.5M)	30mM	0.0048	2.4
MgCl2 (100mM)	2.5mM	0.01	5
GTP (100mM)	1mM	0.004	2
DTT (100mM)	8mM	0.032	16
Smart-seq3xpress TSO (100µM)	0.75µM	0.003	1.5
Maxima H-minus RT enzyme (200U/µL)	2U	0.004	2
Nuclease Free Water	-	0.0325	16.25
Total	-	0.1µL	50µL

Add 0.1 µL RT mix to each well of a 384 well plate.

Replace the storage seal with a PCR seal. Ensure that the plate is properly sealed, to avoid evaporation.

Briefly centrifuge to collect reaction at the bottom.

Incubate the plate in a thermocycler at:

Temp	Time	Cycles
42 °C	90 min	1x
50 °C	2 min	10x
42 °C	2 min	
85 °C	5 min	1x
4 °C	Hold	Hold

Preamplification PCR

Start preparing the PCR mix, when the incubation of the reverse transcription reaction is near completion, by combining the following components.

Reagent	Reaction conc.	µL per reaction	384 well plate (500 rxns)
SeqAmp PCR buffer (2x)	1X	0.5	250
Fwd Primer (100µM)	0.5µM	0.005	2.5
Rev Primer (100µM)	0.5µM	0.005	2.5
SeqAmp DNA polymerase (1.25u/uL)	0.025U/µL	0.02	10
Nuclease Free Water	–	0.07	35
Total	–	0.6µL	300µL

Add 0.6 µL PCR mix to each well of a 384 well plate.

Briefly centrifuge to collect reaction at the bottom.

Incubate the plate in a thermocycler at:

Step	Temp	Time	Cycles
Initial denaturation	98 °C	1 min	1x
Denaturation	98 °C	10 sec	12-16x*
Annealing	65°C	30 sec	
Elongation	72 °C	4 min	
Final Elongation	72 °C	10 min	1x
Hold	4 °C	Hold	

* depending on cell type (reflecting RNA content per cell)

To prepare indexed sequencing libraries from Smart-seq3express cDNA by tagmentation and PCR, please refer to online protocol: https://www.protocols.io/view/smart-seq3xpress-yxmvmk1yng3p/v2.

Quality Control check

Inspect final library concentration and size distribution after tagmentation, PCR, and clean-up, e.g., on an Agilent Bioanalzyer High Sensitivity DNA Analysis chip.

Representative Bioanalyzer image of a successfully tagmented and pooled library using the SEQURNA RNase inhibitor:

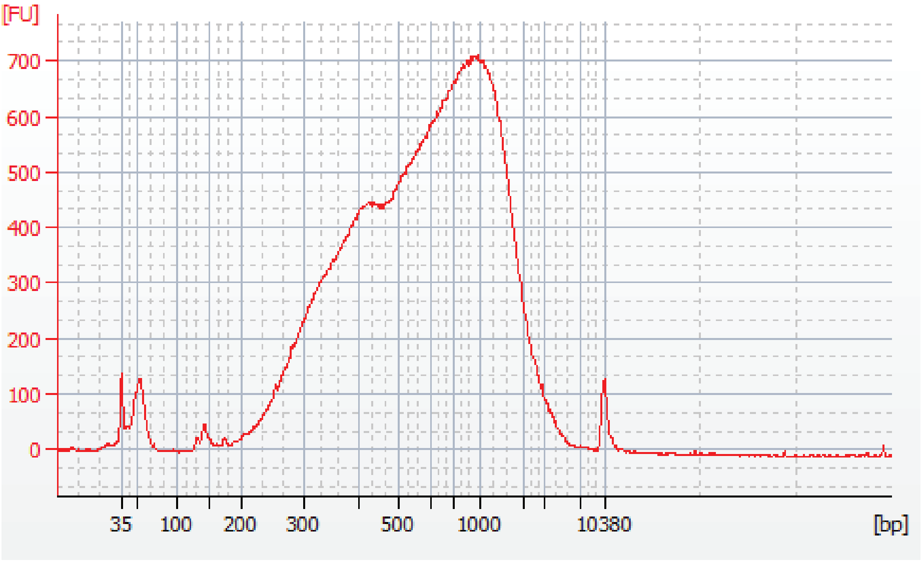

Trace of Smart-seq3express pooled library (after tagmentation and PCR amplification) from sorted HEK cells, using an Agilent Bioanalzyer High Sensitivity DNA Analysis chip.
