# Supplementary material for: Introducing synthetic thermostable RNase inhibitors to single-cell RNA-seq
Source: Nat Commun. 2024 Sep 27;15:8373. doi: 10.1038/s41467-024-52717-4 (PMC11437267; doi:10.1038/s41467-024-52717-4)
Supplement: Supplementary file 3 — Description of Additional Supplementary Files [file 41467_2024_52717_MOESM3_ESM.pdf]

Description of Additional Supplementary Files

Supplementary Data 1. Statistical test results for conditions against controls for selected plots.

Supplementary Data 2. Number of samples for each experiment.
