# Supplementary material for: Introducing synthetic thermostable RNase inhibitors to single-cell RNA-seq
Source: Nat Commun. 2024 Sep 27;15:8373. doi: 10.1038/s41467-024-52717-4 (PMC11437267; doi:10.1038/s41467-024-52717-4)
Supplement: Supplementary file 6 — Reporting Summary [file 41467_2024_52717_MOESM6_ESM.pdf]

nature portfolio

Corresponding author(s): BJÖRN REINIUS

Last updated by author(s): Aug 20, 2024

Reporting Summary

Nature Portfolio wishes to improve the reproducibility of the work that we publish. This form provides structure for consistency and transparency in reporting. For further information on Nature Portfolio policies, see our Editorial Policies and the Editorial Policy Checklist.

Statistics

For all statistical analyses, confirm that the following items are present in the figure legend, table legend, main text, or Methods section.

n/a	Confirmed	
☐	☒	The exact sample size (*n*) for each experimental group/condition, given as a discrete number and unit of measurement
☒	☐	A statement on whether measurements were taken from distinct samples or whether the same sample was measured repeatedly
☐	☒	The statistical test(s) used AND whether they are one- or two-sided<br>*Only common tests should be described solely by name; describe more complex techniques in the Methods section.*
☒	☐	A description of all covariates tested
☒	☐	A description of any assumptions or corrections, such as tests of normality and adjustment for multiple comparisons
☒	☐	A full description of the statistical parameters including central tendency (e.g. means) or other basic estimates (e.g. regression coefficient) AND variation (e.g. standard deviation) or associated estimates of uncertainty (e.g. confidence intervals)
☒	☐	For null hypothesis testing, the test statistic (e.g. *F*, *t*, *r*) with confidence intervals, effect sizes, degrees of freedom and *P* value noted<br>*Give P values as exact values whenever suitable.*
☒	☐	For Bayesian analysis, information on the choice of priors and Markov chain Monte Carlo settings
☒	☐	For hierarchical and complex designs, identification of the appropriate level for tests and full reporting of outcomes
☒	☐	Estimates of effect sizes (e.g. Cohen's *d*, Pearson's *r*), indicating how they were calculated

Our web collection on statistics for biologists contains articles on many of the points above.

Software and code

Policy information about availability of computer code

Data collection	Not relevant/not used in this study.
Data analysis	Computational code generated in this study is available at github.com/reiniuslab/SEQURNA.

For manuscripts utilizing custom algorithms or software that are central to the research but not yet described in published literature, software must be made available to editors and reviewers. We strongly encourage code deposition in a community repository (e.g. GitHub). See the Nature Portfolio guidelines for submitting code & software for further information.

Data

Policy information about availability of data

All manuscripts must include a data availability statement. This statement should provide the following information, where applicable:

- Accession codes, unique identifiers, or web links for publicly available datasets
- A description of any restrictions on data availability
- For clinical datasets or third party data, please ensure that the statement adheres to our policy

Sequencing data generated in this study has been deposited to ArrayExpress (accessions E-MTAB-13873 and E-MTAB-13899 for single-cell and bulk RNA-seq libraries, respectively).

Research involving human participants, their data, or biological material

Policy information about studies with human participants or human data. See also policy information about sex, gender (identity/presentation), and sexual orientation and race, ethnicity and racism.

Reporting on sex and gender	Not relevant/not used in this study.
Reporting on race, ethnicity, or other socially relevant groupings	Not relevant/not used in this study.
Population characteristics	Not relevant/not used in this study.
Recruitment	Not relevant/not used in this study.
Ethics oversight	Not relevant/not used in this study.

Note that full information on the approval of the study protocol must also be provided in the manuscript.

Field-specific reporting

Please select the one below that is the best fit for your research. If you are not sure, read the appropriate sections before making your selection.

☒ Life sciences ☐ Behavioural & social sciences ☐ Ecological, evolutionary & environmental sciences

For a reference copy of the document with all sections, see nature.com/documents/nr-reporting-summary-flat.pdf

Life sciences study design

All studies must disclose on these points even when the disclosure is negative.

Sample size	No sample-size calculation was performed before running the experiments. Sample sizes were selected based our previous experience in scRNAseq methods development.
Data exclusions	Individual sequencing libraries of insufficient read depth were excluded from further analyses as described in the Methods.
Replication	The performance of the SEQURNA RNase inhibitor was tested in three different scRNAseq protocols (Smart-seq2, Smart-seq3, Smart-seq3xpress).
Randomization	Not relevant for the study.
Blinding	No blinding was performed. All samples handled uniformly and in parallel for all conditions when possible.

Reporting for specific materials, systems and methods

We require information from authors about some types of materials, experimental systems and methods used in many studies. Here, indicate whether each material, system or method listed is relevant to your study. If you are not sure if a list item applies to your research, read the appropriate section before selecting a response.

Materials & experimental systems

n/a	Involved in the study	
☒	☐	Antibodies
☐	☒	Eukaryotic cell lines
☒	☐	Palaeontology and archaeology
☐	☒	Animals and other organisms
☒	☐	Clinical data
☒	☐	Dual use research of concern
☒	☐	Plants

Methods

n/a	Involved in the study	
☒	☐	ChIP-seq
☒	☐	Flow cytometry
☒	☐	MRI-based neuroimaging

Eukaryotic cell lines

Policy information about cell lines and Sex and Gender in Research

Cell line source(s)	HEK293 (immortalized human embryonic kidney cells) maintained at the Karolinska Institute. The HEK293 line was originally derived in 1973 by Alex van Ed laboratory.
Authentication	The cell line was confirmed by RNA sequencing, matching the expected transcriptional profile of previous HEK293 datasets.
Mycoplasma contamination	The cells were not tested for mycoplasma.
Commonly misidentified lines (See ICLAC register)	None used in our lab.

Animals and other research organisms

Policy information about studies involving animals; ARRIVE guidelines recommended for reporting animal research, and Sex and Gender in Research

Laboratory animals	Mus musculus, C57BL/6J, CAST/EiJ. Adult.
Wild animals	Not used in this study.
Reporting on sex	Not relevant/not used in this study.
Field-collected samples	Not relevant/not used in this study.
Ethics oversight	Jordbruksverket, Sweden. Ethical permit 17956-2018.

Note that full information on the approval of the study protocol must also be provided in the manuscript.

Plants

Seed stocks	Not relevant/not used in this study.
Novel plant genotypes	Not relevant/not used in this study.
Authentication	Not relevant/not used in this study.
